# Supplementary material for: Talin force coupling underlies eukaryotic cell-substrate adhesion
Source: Nat Commun. 2025 Dec 6;16:10950. doi: 10.1038/s41467-025-67354-8 (PMC12686525; doi:10.1038/s41467-025-67354-8)
Supplement: Supplementary file 1 — Supplementary Information [file 41467_2025_67354_MOESM1_ESM.pdf]

Supplementary Material for

Talin force coupling underlies eukaryotic cell-substrate adhesion

Srishti Rangarajan[1], Lena Espeter[1], Hannes C.A. Drexler[2], Anna Chrostek-Grashoff[1] and Carsten Grashoff[1,*]

[1]University of Münster, Institute of Integrative Cell Biology and Physiology, Münster, D-48149, Germany

[2]Max Planck Institute of Molecular Biomedicine, Mass Spectrometry Unit, Münster, D-48149, Germany

*Correspondence to C.G. (e-mail: grashoff@uni-muenster.de)

This PDF file includes:

Supplementary Figures and Supplementary Figure Legends (1-9)

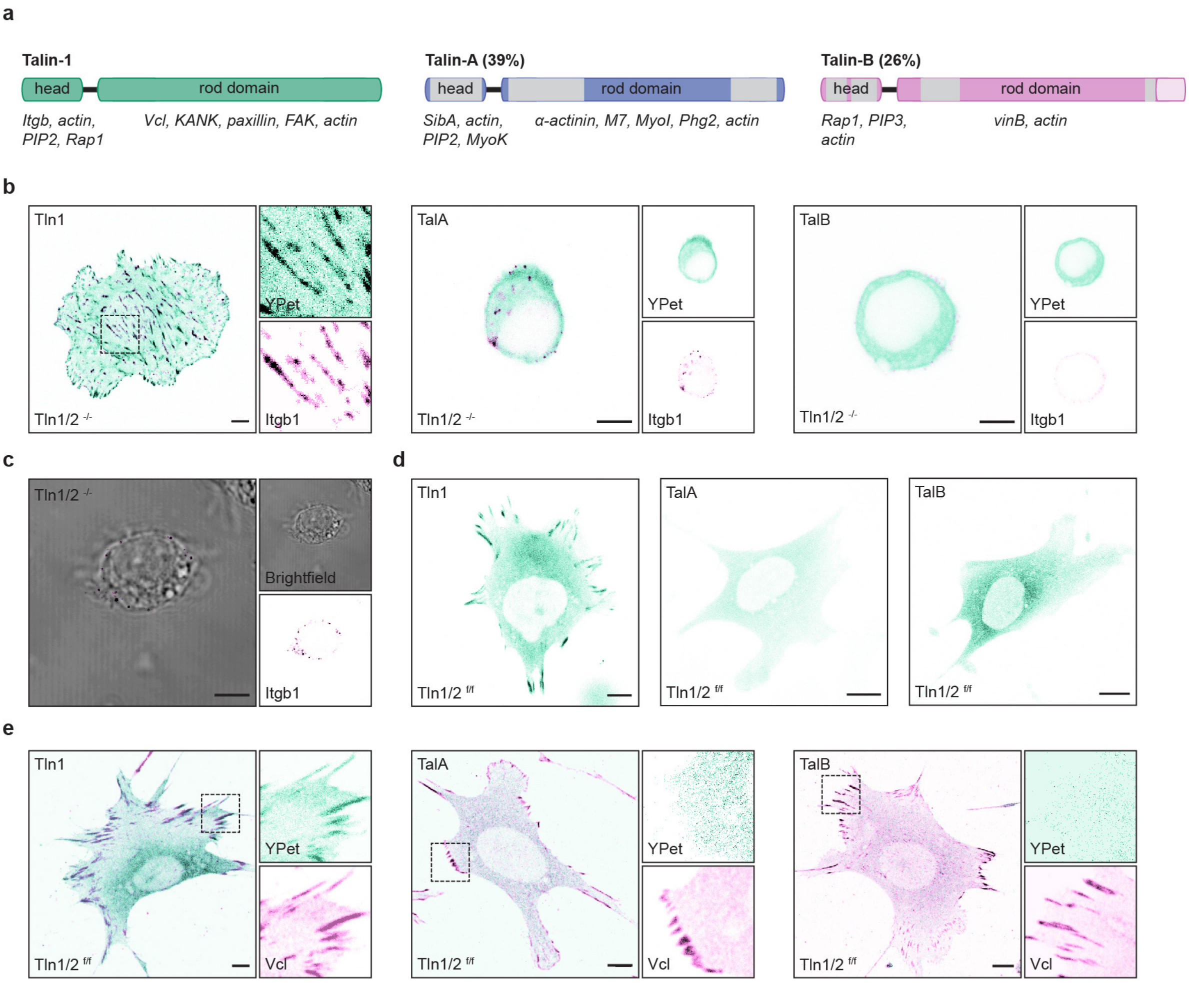

Supplementary figure 1: Amoeboid talin fails to activate integrins and to induce adhesion formation. (**a**) Schematic depiction of the domain structures of Tln1, Tal-A and Tal-B. Percentages indicate the amino acid identify compared to human Tln1, grey areas in TalA and TalB depict regions of high sequence similarity. Indicated below are examples of previously and here identified interaction partners. (**b**) Representative images of Tln1/2$^{-/-}$ cells expressing Tln1, TalA and TalB, C-terminally tagged with YPet. Co-staining with the 9EG7 antibody reveals activation of β1 integrins in Tln1 cells, but not in TalA or TalB expressing cells. (**c**) Representative image of a Tln1/2$^{-/-}$ cell stained with the 9EG7 antibody. Note that these cells are defective in integrin activation[34]. (**d**) Representative images of Tln1/2$^{f/f}$ cells expressing Tln1, TalA and TalB tagged C-terminally with YPet. Note the efficient localization of Tln1 to FAs, whereas TalA and TalB remain cytosolic. (**e**) TalA and TalB are, in contrast to Tln1, not recruited to FAs of Tln1/2$^{f/f}$ cells; FAs were visualized by vinculin co-staining (Vcl). Scale bars, 5 μm in (**b, c**) and 10 μm in (**d, e**).

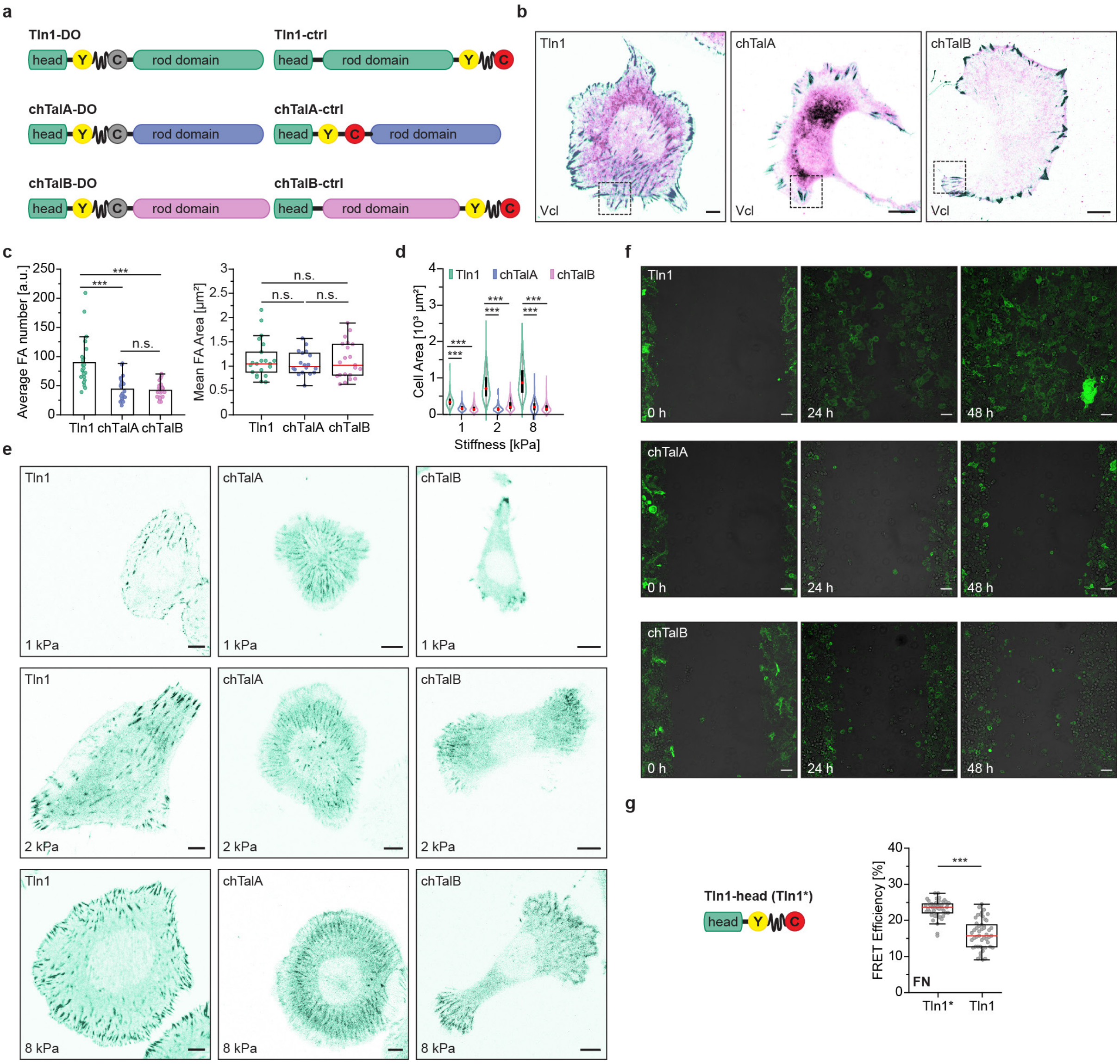

Supplementary figure 2: Cells expressing chTalA and chTalB fail to undergo mesenchymal cell migration. (**a**) Schematic illustrations of control constructs used in FLIM-FRET experiments. The donor only (DO) constructs of Tln1, chTalA and chTalB harbor a point mutation in mCherry and are used to determine the fluorescent lifetime of the donor fluorophore (YPet) in the absence of the acceptor fluorophore (mCherry). Tln1 with a C-terminal HP35 fusion (Tln1-ctrl) is used as a no-force control; chTalA-ctrl harbors a FRET module, in which the mechanosensitive HP35 linker was replaced with a non-stretchable F7 peptide; chTalB with a C-terminal HP35 fusion (chTalB-ctrl) is used as a no-force control in chTalB experiments. (**b**) Representative (uncropped) images of Tln1/2$^{-/-}$ cells expressing Tln1, chTalA and chTalB, co-stained with vinculin (Vcl). The dashed squares indicate the area that is shown as zoom-ins in Fig.1i. The data show that vinculin is efficiently recruited to FAs in all cell lines. (**c**) Quantification of the FA number per cell indicates reduced values in chTalA and

chTalB expressing cells as compared to Tln1 cells. (n=25, 22, 25, N=3). Analysis of the overall FA area between Tln1, chTalA and chTalB cells revealed no significant differences. (n=21, 18, 21; N=3). (**d**) Quantification of the cell area at the indicated stiffness of soft hydrogels. (n=98, 108, 107; 104,133, 117; 104, 98, 120; N=3). (**e**) Representative live-cell images of Tln1, chTalA and chTalB expressing cells cultured on hydrogels of 1 kPa, 2 kPa and 8 kPa. (**f**) Representative live-cell images of Tln1/2$^{-/-}$ cells expressing Tln1, chTalA and chTalB constructs in a wound healing assay. Note that chTalA/B cells are unable to close the wound, even after 48 h. (**g**) Quantification of live-cell FLIM-FRET experiments reveals that deletion of the talin-rod domain leads to significantly increased FRET efficiencies, indicating loss of molecular tension (n=49; 49; N=3).. Statistical significance was determined with a One-way ANOVA (average FA number) and a Two-sample Kolmogorov-Smirnov test (FA area) in (**c**), (**d**) and (**g**); *** $p<0.001$; not significant (n.s.) $p>0.05$. Error bars indicate the SD of the mean. Boxplots show median, 25th and 75th percentile with whiskers reaching to the last data point within 1.5x interquartile range. Scale bars, 10 µm in (**b**), 5 µm in (**e**) and 50 µm in (**f**). Source data and exact p-values are provided in the Source Data file.

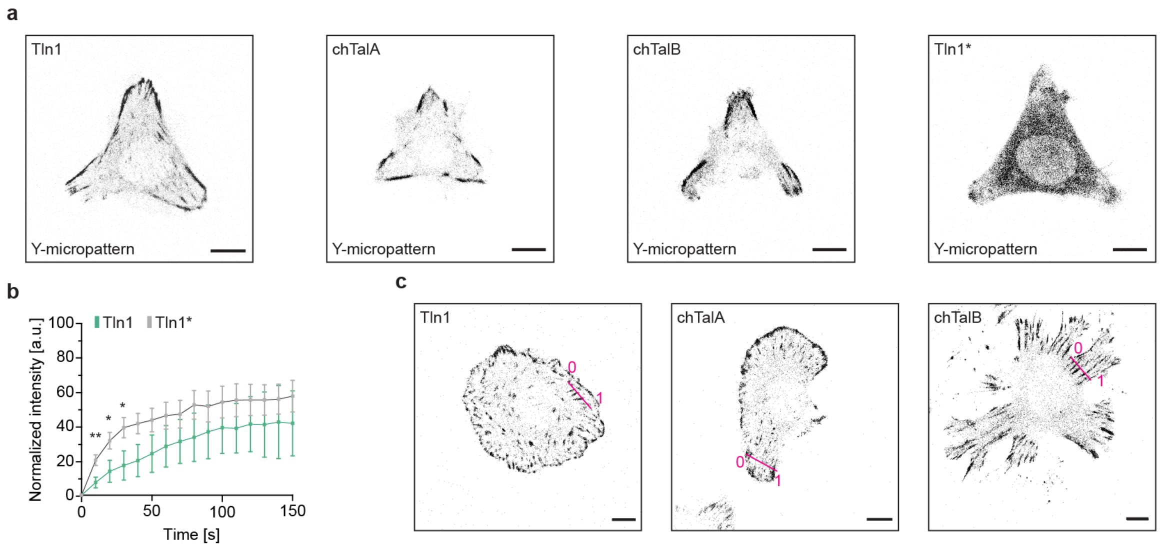

Supplementary figure 3: Analysis of talin turnover and FA dynamics. (**a**) Representative images of cells seeded on FN-coated Y-shaped micropatterns. FRAP assays were performed under these conditions to ensure comparable (i.e., static) cell adhesions. Note that chTalA/B cells were seeded on slightly smaller micropatterns to ensure similar cell morphologies. (**b**) FRAP analysis reveals that the FA recovery rates of the talin-head only construct (Tln1*) are significantly faster compared to full length Tln1. (n=11, 9; N=3). Error bars indicate s.e.m. (**c**) Images of the Tln1, chTalA and chTalB expressing cells used for the kymograph analysis, depicted in Fig. 2b-d. The ROI used for the kymograph analysis is marked in magenta. Statistical significance was determined with a two-sample test for variance. ** $p<0.01$; * $p<0.05$. Scale bars, 10 µm. Source data and exact p-values are provided in the Source Data file.

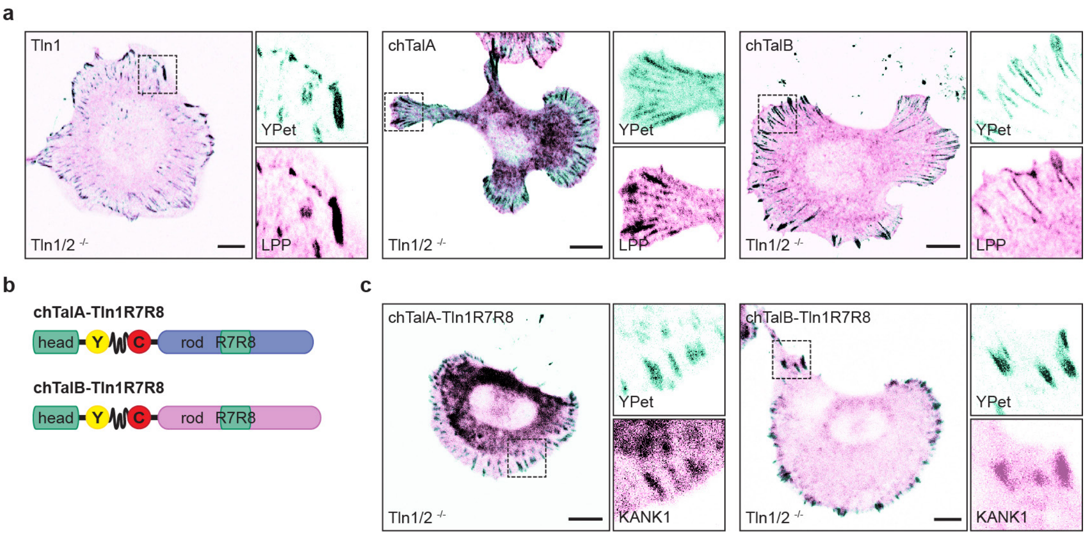

Supplementary figure 4: The amoeboid talin rod domain recruits LPP but not KANK. **(a)** Representative images of Tln1/2$^{-/-}$ cells expressing Tln1, chTalA and chTalB stained with an antibody against Lipoma-preferred-partner (LPP) showing efficient localization of LPP to FAs. (**b**) Schematic illustration of chTalA/B constructs in which the R7/R8 region was replaced with that of Tln1 (chTalA/B–Tln1R7R8). (**c**) Representative images of Tln1/2$^{-/-}$ cells expressing chTalA–Tln1R7R8 and chTalB–Tln1R7R8 showing improved recruitment of KANK1 to FAs. Scale bars, 10 µm.

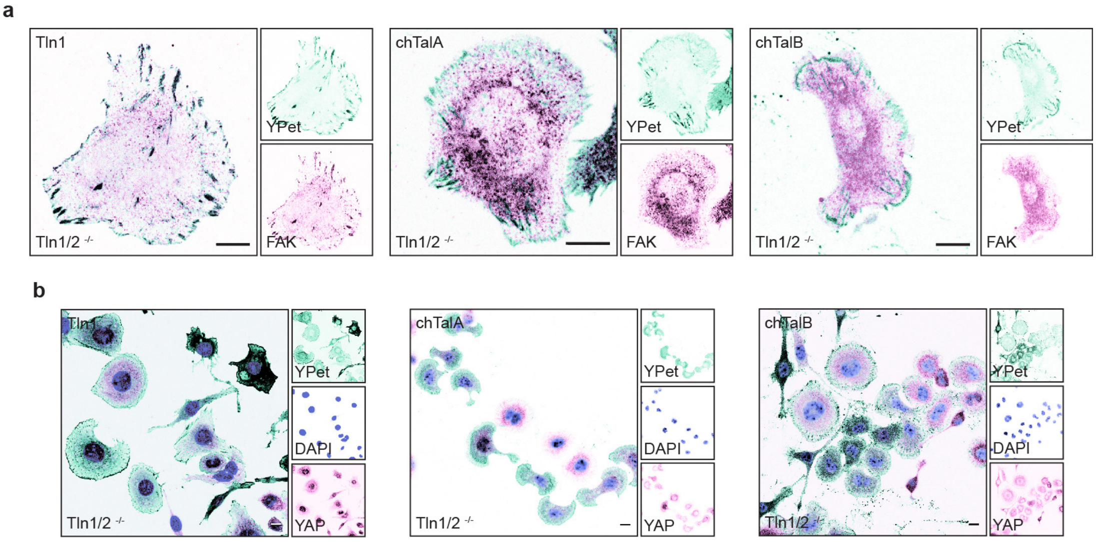

Supplementary figure 5: The amoeboid talin rod domain fails to induce signaling through paxillin, FAK, and YAP. (**a**) Representative images of Tln1/2$^{-/-}$ cells expressing Tln1, chTalA and chTalB (green), stained for total FAK (magenta). Note that FAK is accumulated in the cytoplasm and hardly enriched in FAs of chTalA/B expressing cells. (**b**) Representative overview images of Tln1/2$^{-/-}$ cells expressing Tln1, chTalA and chTalB (green), stained for YAP (magenta) and DAPI (blue). While YAP is efficiently recruited to the nucleus in Tln1 control cells, it is largely cytoplasmic in chTalA/B cells. Scale bar, 10 µm.

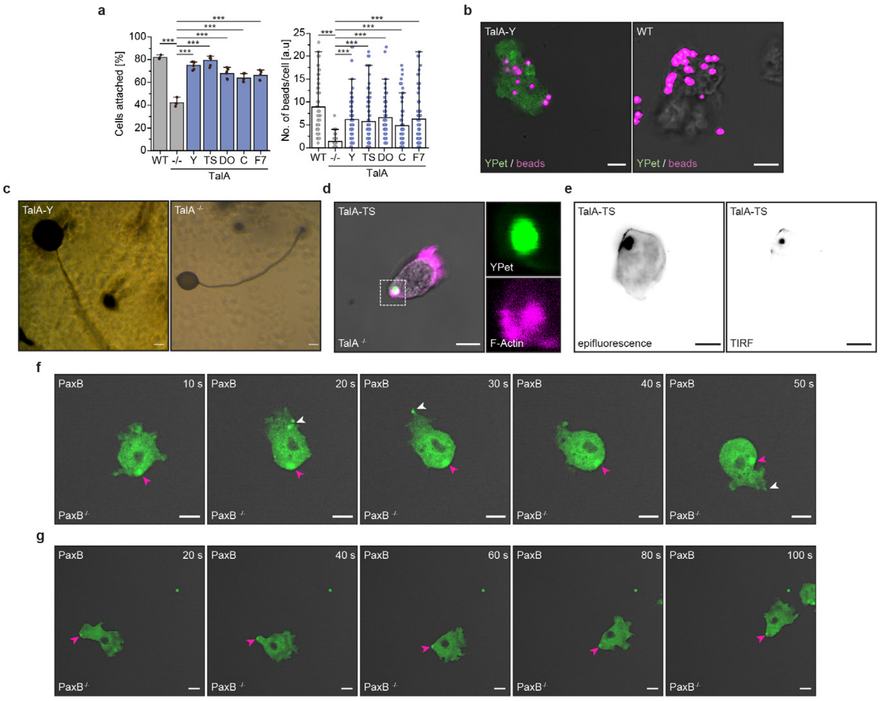

Supplementary figure 6: Characterization of TalA-TS expression in *Dictyostelium* cells. (**a**) Analysis of all generated TalA expressing amoeba. Cell adhesion and phagocytosis defect are rescued by the expression of TalA constructs (N=4, 4, 6, 6, 6, 5, 5); (n=89, 90, 89, 90, 74, 77, 84). (**b**) Representative images of wild type (WT) cells and TalA$^{-/-}$ cells expressing Tal-Y showing phagocytosed microbeads. (**c**) Representative images showing stalk and spore head formation in TalA-Y and TalA$^{-/-}$ expressing amoebae. The data indicate that the insertion of these constructs does not impair *D. discoideum* development, despite constitutive expression of the TlnA protein. (**d**) Representative image of a TalA-TS expressing *Dictyostelium* cell, stained for actin; the merged image is shown in Fig. 4h. Note the co-localization of talin and actin at the dot-like adhesion structure. (**e**) Representative epifluorescence and TIRF image of a fixed TalA$^{-/-}$ cell expressing TalA-TS. The data suggest that the talin-rich structure is in close contact with the underlying substrate. (**f**, **g**) Representative live cell images of a PaxB-deficient *Dictyostelium* cells re-expressing GFP-PaxB[58]. Note that small and transient adhesion-like complexes (white arrowhead) but also dot-like accumulations (magenta arrowhead) of PaxB were observed in a few stationary cells (**f**). Distinct adhesion like complexes were not observed in migrating cells; however, a punctate structure at the proximal end of the cell was frequently seen (**g**). Statistical significance was tested with a two-sample t-test in the cell adhesion assay and with a One-way ANOVA in the phagocytosis assay. Scale bars, 5 µm in (**b**, **d**, **e**, **f**, **g**) and 50 µm in (**c**). Source data and exact p-values are provided in the Source Data file.

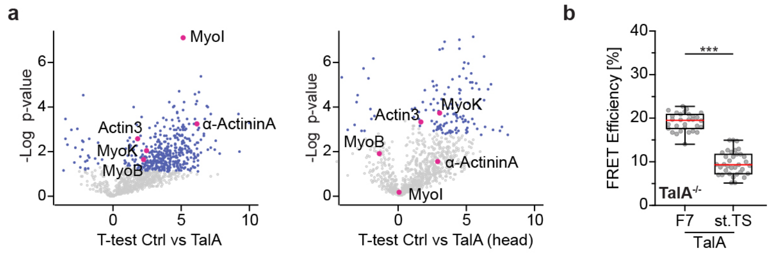

Supplementary figure 7: TalA interactome and high force tension sensor experiment. (**a**) Volcano plots of mass spectrometry data comparing the interactome of full-length TalA (left) and the TalA head domain (right) with that of a control (YPet only, Ctrl). Non-significant proteins are represented in grey whereas statistically significant hits are colored in blue. Note the specific enrichment of actin-associated proteins such as MyoI, MyoK and MyoB but also α-actinin in the full-length TalA samples. (**b**) Live-cell FLIM-FRET analysis of TalA-deficient *Dictyostelium* cells, expressing a no-force FRET control (F7) or an HP35st (st.TS)-based TalA tension sensor detecting force of 9-11 pN[15]. The low FRET efficiency in st.TS samples suggests that TalA in migrating *D. discoideum* cells is exposed to very similar forces as Tln1 in mammalian fibroblasts. (n=34, 33; N=4). Statistical significance was determined with a Two-sample Kolmogorov-Smirnov test, *** p<0.001. Error bars indicate the SD of the mean. Boxplots show median, 25th and 75th percentile with whiskers reaching to the last data point within 1.5x interquartile range. Source data and exact p-values are provided in the Source Data file.

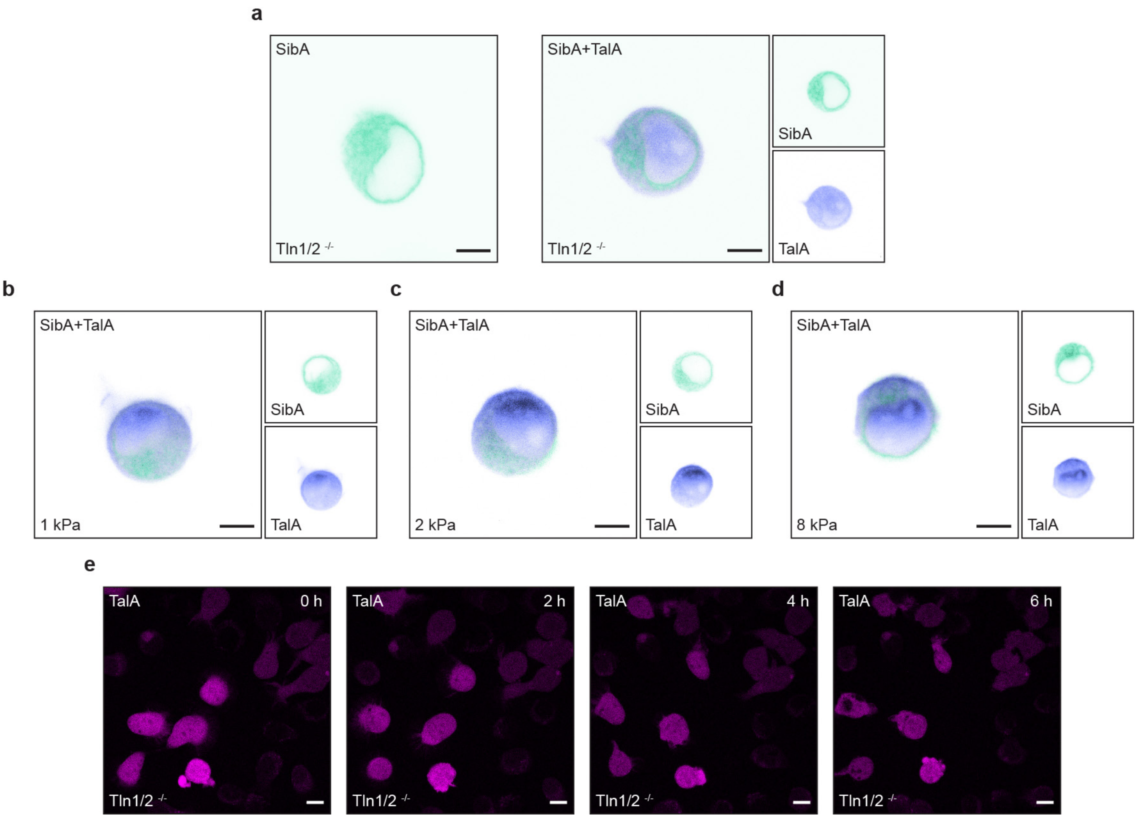

Supplementary figure 8: Exemplary images of non-confined cells used in amoeboid migration assays. (**a**) Representative images of a Tln1/2$^{-/-}$ cells, seeded on FN-coated glass surfaces, expressing SibA only, or SibA and TalA. Note that cells fail to spread and form adhesions in 2D conditions. (**b-d**) Representative images of Tln1/2$^{-/-}$ cells expressing SibA and TalA seeded on soft hydrogels of 1 kPa, 2 kPa, and 8 kPa. Cells fail to spread under all conditions. (**e**) Representative fluorescent image of TalA in confined Tln1/2$^{-/-}$ cells expressing SibA and TalA. A distinct subcellular localization was not detectable. Scale bars, 5 µm (**a-d**) and 10 µm in (**e**).

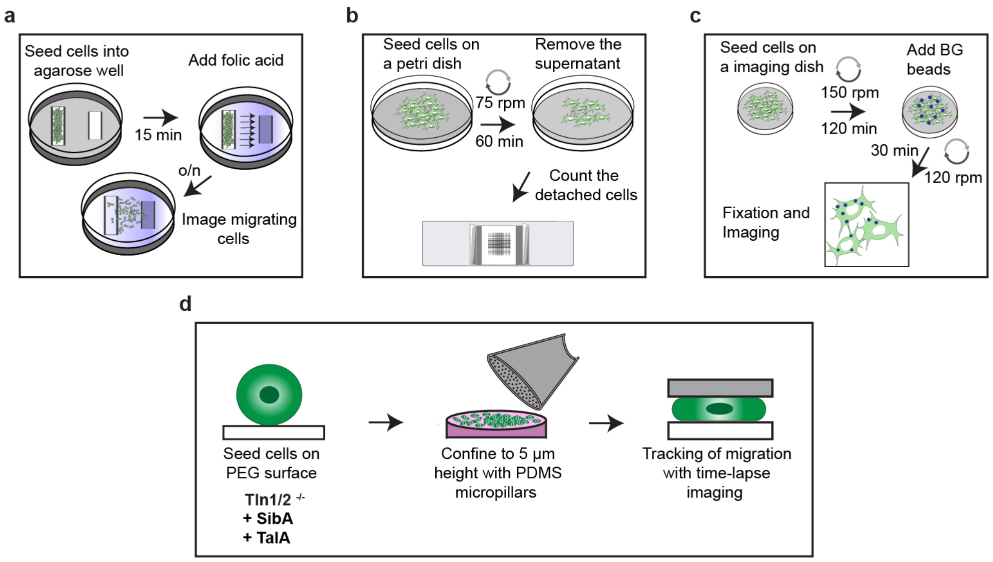

Supplementary figure 9: Schematic illustrations of applied methodologies. (**a**) Depiction of the under-agarose assay used for live cell FLIM-FRET measurements. The cells were allowed to undergo chemotaxis using folic acid as the chemo-attractant. (**b**) Illustration of the performed cell adhesion assay. (**c**) Schematic illustration of the here used phagocytosis assay. (**d**) Schematic depiction of the here used confinement protocol. The PDMS micropillars used for confining the cells were 5 µm in height.
