# Supplementary material for: Talin force coupling underlies eukaryotic cell-substrate adhesion
Source: Nat Commun. 2025 Dec 6;16:10950. doi: 10.1038/s41467-025-67354-8 (PMC12686525; doi:10.1038/s41467-025-67354-8)
Supplement: Supplementary file 2 — Description of Additional Supplementary File [file 41467_2025_67354_MOESM2_ESM.pdf]

Description of Additional Supplementary Files

Supplementary Movie 1 :

Tln1/2-/- fibroblast expressing Tln1. Representative time-lapse movie showing the coordinated assembly and disassembly of FA in control cells. Scale bar, 10 µm.

Supplementary Movie 2:

Tln1/2-/- fibroblast expressing chTalA. Representative time-lapse movie of chTalA cells highlighting the inability to properly disassemble FAs. Scale bar, 10 µm.

Supplementary Movie 3 :

Tln1/2-/- fibroblast expressing chTalB. Representative time-lapse movie of chTalB cells highlighting the inability to properly disassemble FAs. Scale bar, 10 µm.

Supplementary Movie 4:

TalA accumulates at the posterior end of migrating D. discoideum cells. Time-lapse videos of TalA / cells expressing TalA-TS migrating under agarose in a chemotactic gradient of folic acid. Note the posterior localization of TalA accumulating in a dot-like adhesions structure. Scale bar, 5 µm.

Supplementary Movie 5 :

Tln1/2-/- fibroblast under confinement. Representative brightfield video recorded for 12 hours. Cells were seeded on pLL-PEG and subjected to a confinement of 5 µm. Scale bar, 50 µm.

Supplementary Movie 6:

Tln1/2-/- fibroblast expressing SibA under confinement. Representative brightfield video recorded for 12 hours. Cells were seeded on pLL-PEG and subjected to a micropillar confinement height of 5 µm. Note that the expression of SibA does not increase the tendency to undergo amoeboid migration. Scale bar, 50 µm.

Supplementary Movie 7:

Tln1/2-/- fibroblast expressing both, SibA and TalA, under confinement. Representative brightfield video recorded for 12 hours. Cells were seeded on pLL-PEG and subjected to a micropillar confinement height of 5 µm. Note that the expression of SibA and TalA leads to an increased migratory behavior that is distinct from the control conditions. Scale bar, 50 µm.
