# Supplementary material for: Talin force coupling underlies eukaryotic cell-substrate adhesion
Source: Nat Commun. 2025 Dec 6;16:10950. doi: 10.1038/s41467-025-67354-8 (PMC12686525; doi:10.1038/s41467-025-67354-8)
Supplement: Supplementary file 10 — Reporting summary [file 41467_2025_67354_MOESM10_ESM.pdf]

nature portfolio

Corresponding author(s): Carsten Grashoff
Last updated by author(s): November 20, 2025

Reporting Summary

Nature Portfolio wishes to improve the reproducibility of the work that we publish. This form provides structure for consistency and transparency in reporting. For further information on Nature Portfolio policies, see our Editorial Policies and the Editorial Policy Checklist.

Statistics

For all statistical analyses, confirm that the following items are present in the figure legend, table legend, main text, or Methods section.

n/a	Confirmed	
☐	☒	The exact sample size (*n*) for each experimental group/condition, given as a discrete number and unit of measurement
☐	☒	A statement on whether measurements were taken from distinct samples or whether the same sample was measured repeatedly
☐	☒	The statistical test(s) used AND whether they are one- or two-sided<br>*Only common tests should be described solely by name; describe more complex techniques in the Methods section.*
☒	☐	A description of all covariates tested
☐	☒	A description of any assumptions or corrections, such as tests of normality and adjustment for multiple comparisons
☐	☒	A full description of the statistical parameters including central tendency (e.g. means) or other basic estimates (e.g. regression coefficient) AND variation (e.g. standard deviation) or associated estimates of uncertainty (e.g. confidence intervals)
☐	☒	For null hypothesis testing, the test statistic (e.g. *F*, *t*, *r*) with confidence intervals, effect sizes, degrees of freedom and *P* value noted<br>*Give P values as exact values whenever suitable.*
☒	☐	For Bayesian analysis, information on the choice of priors and Markov chain Monte Carlo settings
☒	☐	For hierarchical and complex designs, identification of the appropriate level for tests and full reporting of outcomes
☒	☐	Estimates of effect sizes (e.g. Cohen's *d*, Pearson's *r*), indicating how they were calculated

Our web collection on statistics for biologists contains articles on many of the points above.

Software and code

Policy information about availability of computer code

Data collection	For confocal imaging on Zeiss LSM 880, the ZEN Software 2.3 (black edition) was used. All confocal images were processed in ZEN 2.6 (blue edition). For acquiring FLIM data on the Zeiss LSM 880 system the ZEN software 2.3 (black edition) was used together with the SymPhoTime 64 2.6 software (PicoQuant). FRAP data was collected on a Zeiss LSM 880 using the ZEN Software 2.3 (black edition). For TIRF Imaging, Zeiss Elyra 7 microscope was used and acquisition and processsing was done with ZEN 2.6 (blue edition). Reversed-phase liquid chromatography was performed using a nanoElute 2 UHPLC system by Bruker Daltonics.
Data analysis	FLIM data was analyzed using SymPhoTime 64 2.6 software (PicoQuant). FRAP analysis was performed using FIJI 1.54f software and Jay_Plugins (https://research.stowers.org/imagejplugins/zipped_plugins.html), Excel (Microsoft Office 365), and Origin Pro 2023b (OriginLab). Cell area measurement, wound healing assay, FA quantification and Mander's coefficent calcultions were done using ImageJ. Random migration analysis was done using mTrackJ plugin in ImageJ. For analysis of migration under confinement, TrackMate-StarDist program was used. Cell adhesion assay and phagocytosis were analysed using Excel sheets. Normality test, t-test, One-way Anova and Two-sided Kolmogorov-Smirnov (KS) test were performed using OriginPro 2023b (OriginLab). All graphs were plotted in OriginPro 2023b (OriginLab).

For manuscripts utilizing custom algorithms or software that are central to the research but not yet described in published literature, software must be made available to editors and reviewers. We strongly encourage code deposition in a community repository (e.g. GitHub). See the Nature Portfolio guidelines for submitting code & software for further information.

Data

Policy information about availability of data

All manuscripts must include a data availability statement. This statement should provide the following information, where applicable:

- Accession codes, unique identifiers, or web links for publicly available datasets
- A description of any restrictions on data availability
- For clinical datasets or third party data, please ensure that the statement adheres to our policy

The data and statistical evaluations supporting the findings of this study are available within the article, the Supplementary Information, and the Source Data file. Mass spectrometry data generated in this study have been deposited at the ProteomeXchange Consortium via the PRIDE partner repository (ProteomeXchange accession: PXD062078).

Research involving human participants, their data, or biological material

Policy information about studies with human participants or human data. See also policy information about sex, gender (identity/presentation), and sexual orientation and race, ethnicity and racism.

Reporting on sex and gender	n.a.
Reporting on race, ethnicity, or other socially relevant groupings	n.a.
Population characteristics	n.a.
Recruitment	n.a.
Ethics oversight	n.a.

Note that full information on the approval of the study protocol must also be provided in the manuscript.

Field-specific reporting

Please select the one below that is the best fit for your research. If you are not sure, read the appropriate sections before making your selection.

☒ Life sciences ☐ Behavioural & social sciences ☐ Ecological, evolutionary & environmental sciences

For a reference copy of the document with all sections, see nature.com/documents/nr-reporting-summary-flat.pdf

Life sciences study design

All studies must disclose on these points even when the disclosure is negative.

Sample size	All experiments were performed on 3-6 independent days to ensure reproducibility. Shown immunostainings and live cell images are representative of at least 3 independent experiments. In case of FLIM experiments, 30-45 individual cells were recorded. In case of FRAP experiment, 15 cells were analyzed. For area calculations, migration analysis, around 200-300 cells were analysed. For phagocytosis, 100 cells were imaged. For co-localisation and FA quantification, 15 cells were measured. Formal sample size calculation was not performed. Instead, sample size for FLIM experiments was based on previous publications (Austen et al. NCB, 2015; Ringer et al. Nature Methods, 2017) showing that biological effects can be documented and statistically evaluated with the chosen number of cells. Sample size was kept similar between experimental conditions.
Data exclusions	FLIM data analysis in mammalian cells was restricted to the focal adhesion signal as described in Material and Methods. Images with insufficient signal intensity were excluded manually from the analysis. Data exclusion criteria were pre-established.
Replication	All cell-experiments were repeated independently on at least for 3 independent experimental days.
Randomization	Samples were not randomized, as the construct expression correlates with an obvious cellular phenotype.
Blinding	For data acquisition and analysis the investigators were not blinded as the phenotype of transfected cells lines were so obvious that blinding was impossible and inappropriate.

Reporting for specific materials, systems and methods

We require information from authors about some types of materials, experimental systems and methods used in many studies. Here, indicate whether each material, system or method listed is relevant to your study. If you are not sure if a list item applies to your research, read the appropriate section before selecting a response.

Materials & experimental systems

n/a	Involved in the study	
☐	☒	Antibodies
☐	☒	Eukaryotic cell lines
☒	☐	Palaeontology and archaeology
☐	☒	Animals and other organisms
☒	☐	Clinical data
☒	☐	Dual use research of concern
☒	☐	Plants

Methods

n/a	Involved in the study	
☒	☐	ChIP-seq
☒	☐	Flow cytometry
☒	☐	MRI-based neuroimaging

Antibodies

Antibodies used

primary antibodies:
anti-talin (TA205, Bio-Rad, MCA725G), anti-vinculin (Sigma-Aldrich, V9131), anti-p-S19-myosin light chain 2 (Cell Signaling, 3671), anti-LPP (Abcam, ab126608), anti-tubulin (DM1A) (Sigma-Aldrich, T6199), anti-FAK (Millipore, 06-543), anti-pY397-FAK (Invitrogen, 44-624), anti-Paxillin (BD Transduction Laboratories, 610051), anti-pY118-Paxillin (Millipore, 07-1440), anti-GFP (Abcam, ab290),anti-YAP (63.7) (Santa Cruz, sc-101199), anti-Integrin β1 (9EG7) (BD Pharmingen, 550531)
secondary antibodies:
anti-mouse IgG Alexa Fluor 647 (Thermo Fisher Scientific, A21235), anti-rabbit IgG Alexa Fluor 647 (Thermo Fisher Scientific, A21244), anti-mouse IgG Alexa Fluor 405 (Thermo Fischer Scientific, A48255), anti-rat IgG Alexa Fluor 647 (Thermo Fischer Scientific, A21247) anti-mouse IgG HRP (Bio-Rad, 170-6516), anti-rabbit IgG HRP (Bio-Rad, 170-6515).
Other staining reagent:
Alexa Fluor 647 Phalloidin (Thermo Fisher Scientific, A22287), DAPI (Sigma, D8417).

Validation

anti-talin (TA205, mouse monoclonal, Bio-Rad, MCA725G; WB: 1:2000)
application(s): flow cytometry, immunofluorescence, Immunohistology, Immunoprecipitation, Western blotting
species reactivity: human, rabbit, chicken
specificity: human Talin
anti-vinculin (mouse monoclonal, Sigma-Aldrich, V9131; IF: 1/400)
application(s): immunohistochemistry, indirect immunofluorescence, Western blot
species reactivity: bovine, canine, mouse, rat, turkey, human, chicken, frog
specificity:Reacts strongly with human vinculin. Shows cross-reactivity with smooth muscle metavinculin.
anti-p-S19-myosin light chain 2 (rabbit polyclonal, Cell Signaling, 3671; IF: 1:50)
application(s): Western Blotting, Immunofluorescence
species reactivity: human, mouse, Rat, Drosophila melanogaster
specificity: myosin light chain 2 (smooth muscle) only when phosphorylated at serine 19. The antibody does not crossreact with the cardiac isoform of myosin light chain 2.
anti-LPP (rabbit Monoclonal, Abcam, ab126608; IF: 1:200)
application(s): immunohistochemistry, western blot
species reactivity: human, mouse, rat
specificity: Anti-LPP
anti-tubulin (DM1A) (mouse monoclonal, Sigma-Aldrich, T6199; WB: 1/10000)
application(s): immunocytochemistry , immunohistochemistry, immunoprecipitation, western blot
species reactivity: yeast, mouse, amphibian, human, rat, chicken, fungi, bovine
specificity: Anti-α-Tubulin antibody. The antibody reacts best with chicken fibroblasts.
anti-FAK (rabbit polyclonal, Millipore, 06-543; IF: 1:200, WB: 1:2000)
application(s): immunocytochemistry, immunoprecipitation, western blot
species reactivity: human, mouse, rat, hamster
specificity: Anti-FAK with RIPA buffer lysates
anti-pY397-FAK (rabbit monoclonal, Invitrogen, 44-624; IF: 1:200, WB: 1:1000 in 5% BSA)
application(s): western blot
species reactivity: Chicken, Fruit fly, Human, Mouse, Rat, Xenopus
specificity: cross-reacts with the autophosphorylation site on Proline-rich/Ca2+- activated tyrosine kinase (Pyk2).
anti-Paxillin (mouse Monoclonal, BD Transduction Laboratories, 610051; IF: 1:200; WB: 1:1000
application(s): immunocytochemistry , immunohistochemistry, immunoprecipitation, western blot
species reactivity: Chicken, dog, Human, Mouse, Rat
specificity: anti paxillin
anti-pY118-Paxillin (rabbit Polyclonal, Millipore, 07-1440; IF-1:200; WB:1:1000)
application(s): western blot
species reactivity: Chicken, dog, Human, Mouse, Rat, Pig, Xenopus
specificity: Specific for Tyr118
anti-GFP (rabbit polyclonal Abcam, ab290; WB: 1:2000)
application(s): immunocytochemistry , immunohistochemistry, immunoprecipitation, western blot, ELISA
species reactivity: all species
specificity: detection of all variants of Aequorea victoria GFP, including RFP, YFP, CFP, and EGFP
anti-YAP (63.7) (mouse monoclonal, Santa Cruz, sc-101199; IF:1:400)
application(s): immunocytochemistry , immunohistochemistry, immunoprecipitation, western blot, ELISA

species reactivity: mouse, rat, human
specificity: YAP in HeLa nuclear lysates
anti-Integrin β1 (9EG7) (rat monoclonal, BD Pharmingen, 550531; IF: 1:200)
application(s): immunocytochemistry , immunohistochemistry, immunoprecipitation
species reactivity: mouse, rat
specificity: specific for beta1 integrin
anti-mouse IgG Alexa Fluor 647 (polyclonal, Thermo Fisher Scientific, A21235; 1:200)
application(s): Immunohistochemistry; Immunocytochemistry, Flow Cytometry
species reactivity: mouse
anti-rabbit IgG Alexa Fluor 647 (polyclonal, Thermo Fisher Scientific, A21244; 1:200)
application(s):Immunocytochemistry, Flow Cytometry
species reactivity: rabbit
anti-mouse IgG Alexa Fluor 405 (polyclonal, Thermo Fischer Scientific, A48255; 1:500)
application(s): Immunocytochemistry
species reactivity: mouse
anti-rat IgG Alexa Fluor 647 (polyclonal, Thermo Fischer Scientific, A21247; 1:500)
application(s): Western blot, immunoprecipitation, Immunohistochemistry; Immunocytochemistry
species reactivity: rat
anti-mouse IgG HRP (polyclonal, Bio-Rad, 170-6516; WB: 1/10000)
recommended dilution: 1:3000
anti-rabbit IgG HRP (polyclonal, Bio-Rad, 170-6515; WB: 1/10000)
recommended dilution: 1:3000

Eukaryotic cell lines

Policy information about cell lines and Sex and Gender in Research

Cell line source(s)	AmphoPack 293 cell line (Clonetech - Takara Bio Europe, 631505). Talin1/2(-/-) and Talin(f/f) mouse fibroblast cells described in Austen.et.al, NCB 2015 and Theodosiou et al, eLife, 2016.
Authentication	The AmphoPack 293 cell line was purchased from the indicated supplier and was not further authenticated. Fibroblast cells were not further authenticated.
Mycoplasma contamination	Cells were not tested for mycoplasma contamination.
Commonly misidentified lines (See ICLAC register)	No commonly misidentified cell line was used.

Animals and other research organisms

Policy information about studies involving animals; ARRIVE guidelines recommended for reporting animal research, and Sex and Gender in Research

Laboratory animals	Dictyostelium discoideum AX2 - gift from Jan Faix, VinA(-/-) - gift from Gunther Gerisch, DH1-10 - gift from Pierre Cosson, TalA(-/-), SibA(-/-), AX3 Pax(-/-)-GFP - from DictyBase
Wild animals	n.a.
Reporting on sex	n.a.
Field-collected samples	n.a.
Ethics oversight	n.a.

Note that full information on the approval of the study protocol must also be provided in the manuscript.

Plants

Seed stocks	n.a.
Novel plant genotypes	n.a.
Authentication	n.a.
